# No Evidence for Pre-Copulatory Sexual Selection on Sperm Length in a Passerine Bird

**DOI:** 10.1371/journal.pone.0032611

**Published:** 2012-02-27

**Authors:** Jan T. Lifjeld, Terje Laskemoen, Oddmund Kleven, A. Tiril M. Pedersen, Helene M. Lampe, Geir Rudolfsen, Tim Schmoll, Tore Slagsvold

**Affiliations:** 1 Natural History Museum, National Centre for Biosystematics (NCB), University of Oslo, Oslo, Norway; 2 Department of Ecology and Natural Resource Management, Norwegian University of Life Sciences, Ås, Norway; 3 Department of Biology, Centre for Ecological and Evolutionary Synthesis (CEES), University of Oslo, Oslo, Norway; 4 Department for Environmental Radioactivity, Norwegian Radiation Protection Authority, Tromsø, Norway; 5 Evolutionary Biology, University of Bielefeld, Bielefeld, Germany; German Primate Centre, Germany

## Abstract

There is growing evidence that post-copulatory sexual selection, mediated by sperm competition, influences the evolution of sperm phenotypes. Evidence for pre-copulatory sexual selection effects on sperm traits, on the other hand, is rather scarce. A recent paper on the pied flycatcher, *Ficedula hypoleuca*, reported phenotypic associations between sperm length and two sexually selected male traits, i.e. plumage colour and arrival date, thus invoking pre-copulatory sexual selection for longer sperm. We were unable to replicate these associations with a larger data set from the same and two additional study populations; sperm length was not significantly related to either male plumage colour or arrival date. Furthermore, there was no significant difference in sperm length between populations despite marked differences in male plumage colour. We also found some evidence against the previously held assumption of longer sperm being qualitatively superior; longer sperm swam at the same speed as shorter sperm, but were less able to maintain speed over time. We argue that both empirical evidence and theoretical considerations suggest that the evolution of sperm morphology is not primarily associated with pre-copulatory sexual selection on male secondary sexual traits in this or other passerine bird species. The relatively large between-male variation in sperm length in this species is probably due to relaxed post-copulatory sexual selection.

## Introduction

Sexual selection promotes the evolution of traits that increase an individual's chance in getting access to mates or enhance the probability of fertilization after mating. The two processes are often distinguished as pre- and post-copulatory sexual selection [1] for internal fertilizers. During the past four decades, evolutionary biologists have uncovered that sperm competition is frequent across the animal kingdom [2]. Post-copulatory sexual selection has therefore been increasingly recognized as an important evolutionary process [1], in addition to the more classical form of behaviour-based, pre-copulatory sexual selection. Post-copulat5ory sexual selection is particularly relevant for the evolution of sperm phenotypes and their performance in sperm competition [3]. For example, sperm competition theory can be used to explain variation in sperm size across species. On the one hand, sperm competition can be seen as a raffle which selects for numerous and tiny sperm [4]. On the other hand, sperm competition might select for longer sperm if sperm size is positively associated with competitive fertilizing ability, for example through increased swimming speed or longevity of longer sperm [5], [6]. Comparative evidence suggests that sperm competition actually selects for shorter sperm in some groups and longer sperm in others [6]. Explanations based on post-copulatory sexual selection thus contribute to our understanding of the huge sperm size heterogeneity observed across the animal kingdom.

There is also accumulating evidence for selection by sperm competition at the intra-specific level. In passerine birds, between-male variation in sperm length is lower in species with a higher risk of sperm competition [7]–[9]. This is consistent with sperm competition acting as a stabilizing selection pressure. But how then is intra-specific variation in sperm length shaped in species with relaxed sperm competition? Is the variation selectively neutral or can pre-copulatory sexual selection be involved? It is a well-established fact that many traits subject to pre-copulatory sexual selection maintain high phenotypic variance [10]. A recent study of the pied flycatcher *Ficedula hypoleuca* is unique in suggesting a role of pre-copulatory sexual selection on sperm length [11]. Calhim *et al.* [11] found a positive correlation between sperm length and two male traits preferred by females in social mate choice; black plumage and early arrival. As this species is characterized by relaxed sperm competition, at least in northern Europe [12]–[16], Calhim *et al.* [11] suggested that there is pre-copulatory sexual selection for longer sperm. Previous studies have found that female preferences for black and early arriving males may evolve through direct benefits in social mate choice, such as better nest sites or territories [17], [18] and better paternal care [19]. An implication of Calhim *et al.*'s [11] finding is that females might also benefit though insured fertility by copulating with males with longer and presumably more fertile sperm. This would be in support of the phenotype-linked fertility hypothesis [20]. Alternatively, females might benefit indirectly through the production of sons that inherit their father's high-quality sperm traits (the “sexy sperm” hypothesis; [21]).

Calhim *et al.*'s [11] study was based on a relatively small sample of males (*N* = 17 and *N* = 13 for plumage colour and arrival time, respectively). We wanted to replicate the study with a larger sample from the same study population in Norway, as well as another Norwegian population and one in Germany. In Norway, most male pied flycatchers have a black dorsal plumage, whereas in Central Europe most are brown [22], [23]. Brown morphs in Norway may thus be due to gene flow from the south [23]. If males in Central Europe have shorter sperm than those in Norway, it may explain the correlation found by Calhim *et al.* [11]. We also collected data on sperm velocity from one of the Norwegian populations to test whether longer sperm swim faster, which might indicate higher fertilization efficiency [24], [25].

## Methods

Sperm samples were collected from 80 male pied flycatchers during the breeding seasons of 2009 and 2010. The sampling areas were Oslo, Norway (59°59′N, 10°38′E; i.e. the same study population as in Calhim *et al.* [11]), Røros, Norway (62°28′N, 11°41′E), and Lingen, Germany (52°27′N, 7°15′E). The field work was conducted in adherence with the Norwegian and German regulations for the use of animals in research, which require national permits for trapping, handling (including non-destructive sampling of feces or semen), and ringing wild birds. Such permits were issued by the Directorate for Nature Management, Trondheim, Norway, to JTL (A-license no. 159), HML (A-license no. 199), and TSl (A-license no. 1035), and by Vogelwarte Helgoland, Wilhelmshaven, Germany, to TSc (license no. DEW-0772). The field procedures of sperm collection and sample preservation for sperm morphometrics and motility analysis are described elsewhere [26], [27]. Briefly, all sperm samples (1–3 µl) were fixed in 5% formalin solution for subsequent morphometric analysis using a Leica light microscope with digital imaging software. We measured (±0.1 µm) the length of head, midpiece and tail separately for individual sperm, which sum up to the sperm's total length. We measured 10 sperm cells per male in the Røros and Lingen populations, and 5–30 (mean = 22) sperm per male in the Oslo population, and calculated the mean sperm length and s.d. for each male. Measurement repeatability was calculated for 18 sperm cells from one male measured twice, and the repeatability [28] was high (*r* = 0.97, *F_17,18_* = 57.0, *p*<0.001). For analysis of sperm motility, we made video recordings of fresh sperm samples (Røros population) in the field. Sperm samples were diluted in Dulbecco's Modified Eagle Medium and kept at 40°C. We made one recording within 1 min of sperm sampling, and a second recording after 10 min, to assess how well short and long sperm maintained motility over time. For eight males, the second motility recording, and hence the calculation of relative change in motility, was discarded due to too few motile cells (<20). Each recording contained four to eight non-overlapping view fields on the slide. Videos were analysed using the Hamilton-Thorne CEROS sperm tracker software. Sperm motility was calculated for single sperm as the curvilinear velocity (VCL, in µm s^−1^) with the same settings as described elsewhere [26], [27]. The repeatability of VCL measurements between multiple view fields of the same recording was moderate (*r* = 0.32, *F_18,94_* = 3.79, *p*<0.001). We used the mean VCL of all sperm tracked per recording in our analyses.

Male dorsal plumage coloration was scored visually according to Drost's [29] seven-points scale in the black-to-brown continuum (1 = jet black, 4 = equal amounts of black and brown feathers, 7 = pure brown). Plumage scoring of birds from the Røros and Lingen populations was performed by JTL from digital photos of the bird's dorsal side, and from which the black and brown feathers were easily distinguishable. The plumage of birds from the Oslo population was scored by HML and TSl directly when handled in the field. Arrival date (Oslo population only) was scored as the first day of observation of a male in a territory and as the day the first egg was laid (the latter for comparison with [11]).

The relationships between sperm length and the two sexually selected traits were analysed in mixed-effects models with ‘Population’ and ‘Individual’ as random factors, and either ‘Plumage colour’, ‘Arrival date’ or ‘First egg date’ as the fixed factor. We explicitly examined if ‘Sperm length’ varied among populations. Consequently, we followed guidelines in Gelman and Hill [30] and ran both a model where each population could vary both in terms of intercept and slope, and a simpler model where population could only vary in their intercept. Model fitting and estimates were obtained with linear mixed-effects (lmer) package lme4 in R (version 2.11.0, R Development Core Team, 2007) using restricted maximum likelihood estimates (REML). We followed Baayen *et al.* [31] and used the ‘anova’ function in the lme4 package to compare the quality of the fit between models. Model fit and the significance of including the varying slope parameters were tested using AIC (Akaike's Information Criterion) and log-likelihood ratio statistics (LLR λ^2^) [32]. We used the pvals.fnc function in the R package to compute *p*-values and 95% highest posterior density credible intervals (HPD) for the fixed effect of ‘Sperm length’ on the basis of a Markov Chain Monte Carlo (MCMC) sample with 10,000 simulations when a model with constant slope was chosen. Although the pvals.fnc function reports *p*-values based on both the *t*-statistic and on MCMC procedures, we only report the MCMC *p*-value. Finally, the model fit was checked using visual examination of normal probability plots and residual plots.

## Results

**Figure 1:**
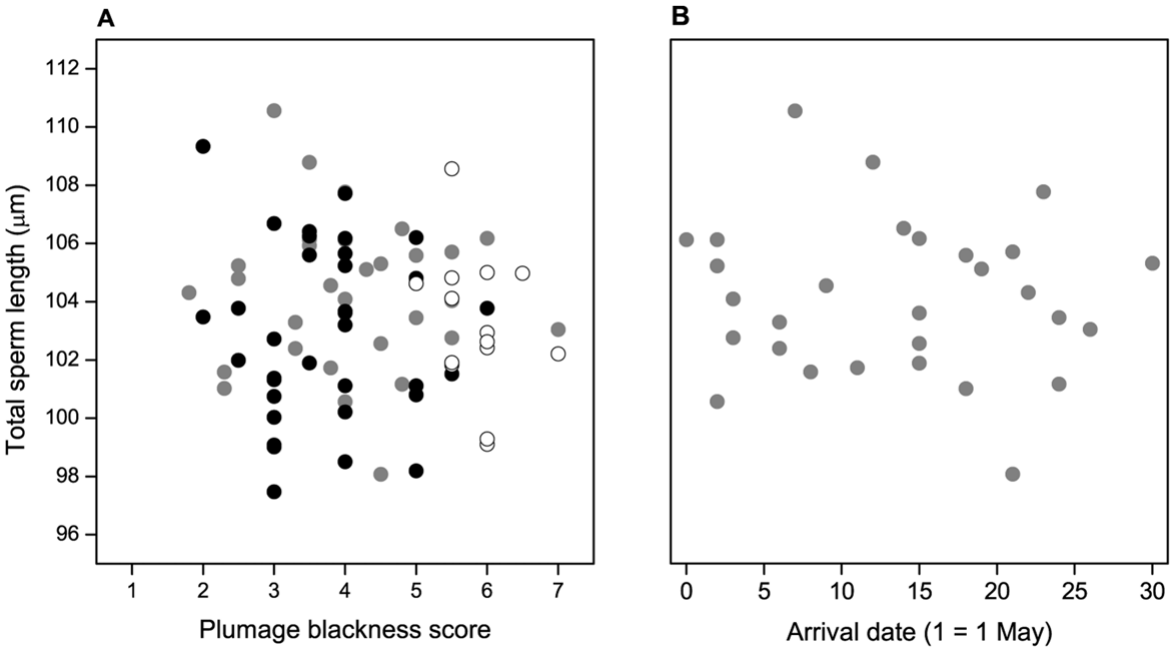
Relationship between sperm length and two sexually selected traits in pied flycatchers. The figure illustrates the lack of associations between a male's average sperm length and his (A) dorsal plumage coloration (black dots (N = 32): Oslo, Norway, grey dots (N = 34): Røros, Norway, open dots (N = 14): Lingen, Germany) and (B) arrival date (Oslo, Norway). Plumage colour scores refer to the 7-point ‘Drost’ scale [23] for the amount of brown versus black in the dorsal plumage, where 1 = jet black, 4 = equal amounts of black and brown, and 7 = pure brown.

Sperm lengths did not differ among the three study populations, but males had a browner dorsal plumage in Germany than in Norway (Table 1). However, there was no significant correlation between plumage colour and sperm length in the total data set (mean Markov Chain Monte Carlo [MCMC] estimate = −0.1581, highest posterior density [HPD] 95% interval: −0.5485, 0.2037, *t* = −0.56, *p* = 0.55; Fig. 1A). A model with population-specific slopes did not make a better fit (LLR λ^2^_2_ = 0.057, *p* = 0.97). Neither were there any significant correlations in the Oslo population with arrival date (mean MCMC estimate = −0.0223, HPD 95% interval: −0.1066, 0.0585, *t* = −0.40, *p* = 0.59; Fig. 1B) or first egg date (mean MCMC estimate = −0.0775, HPD 95% interval: −0.2639, 0.1283, *t* = −0.51, *p* = 0.42).

**Table 1:** Sperm length and male plumage colour in three populations of the pied flycatcher.

Trait	Oslo, Norway (*N* = 32 males)			Røros, Norway (*N* = 34 males)			Lingen, Germany (*N* = 14 males)			ANOVA
Sperm total length (µm)	104.2	±	2.5	102.9	±	3.0	103.2	±	2.5	*F_2,77_* = 1.92, *p* = 0.15
Male plumage colour	4.1	±	1.2	3.7	±	1.0	5.9	±	0.5	*F_2,77_* = 23.2, *p*<0.001

Values are given as mean ± SD, plumage was scored following the Drost scale [29], where 1 = jet black, 4 = equal mix of black and brown feathers, and 7 = pure brown.

Replicate studies should preferably test for a difference in effect sizes [33]. The effect size of plumage colour on sperm length for the Oslo population (Pearson *r* = −0.09, *N* = 32) was significantly smaller than that reported in the Calhim *et al.* [11] study (*r* = −0.73, *N* = 17): Cohen's *q* = 0.84, *Z* = 2.58, *p* = 0.01 [33]. The effect size of laying date on sperm length (*r* = −0.08, N = 21) was also smaller than that reported in the previous study (*r* = −0.63, *N* = 13), although not significantly so at the 5% level (Cohen's *q* = 0.66, *Z* = 1.67, *p*<0.10). Test results were similar (Cohen's *q* = 0.66, *Z* = 1.77, *p*<0.10) when using data on arrival date (*r* = −0.08, *N* = 29) instead of laying date. Hence, our study failed to replicate the two main relationships found by Calhim *et al.* [11] in the same study population.

**Figure 2:**
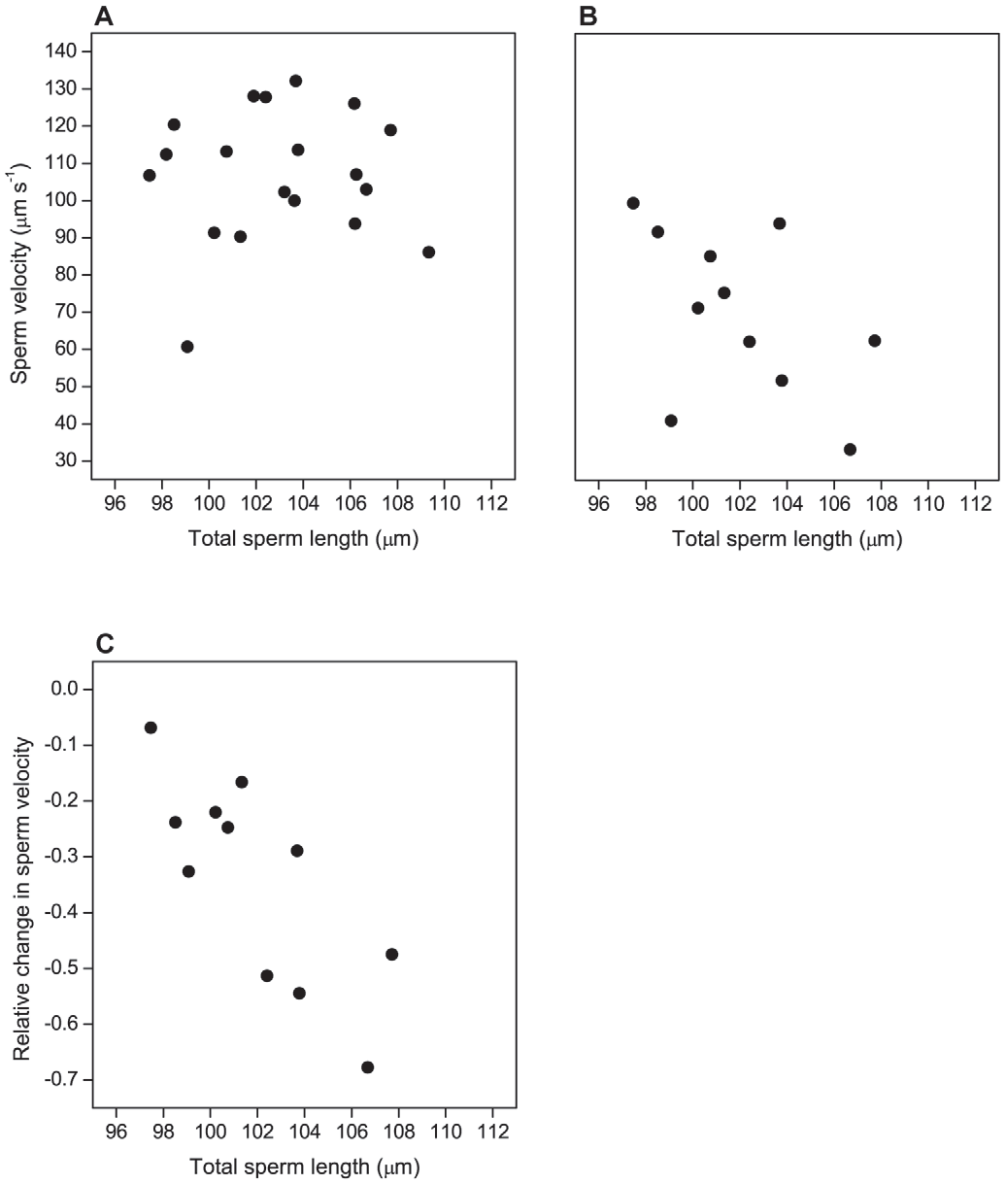
Sperm velocity as a function of sperm length in pied flycatchers. The figure shows the relationship between sperm length and (A) sperm velocity recorded 1 min after ejaculate sampling (*N* = 19, *r* = 0.06, *p* = 0.82), (B) sperm velocity recorded after 10 min (*N* = 11, *r* = −0.50, *p* = 0.12), and (C) relative change in sperm velocity from 1 to 10 min after sampling (*N* = 11, *r* = −0.79, *p* = 0.004). All data from the Røros population, Norway. Note that sample size is smaller than for sperm length (Fig. 1, Table 1) because some ejaculates contained too much noise particles (faeces) for motility analysis.

Sperm length did not explain any significant proportion of the variation among males in sperm velocity recorded immediately after ejaculate sampling (Fig. 2A) or after 10 min of incubation (Fig. 2B). However, males with longer sperm had a larger relative decrease in sperm velocity after 10 min (Fig. 2C).

## Discussion

Our results revealed no significant association between sperm length and two sexually selected male traits, i.e. plumage colour and arrival date, in the pied flycatcher. Nor did the German population have shorter sperm than those in Norway, despite a marked difference in male plumage colour. Hence, we found no empirical support for pre-copulatory sexual selection on sperm length in the pied flycatcher. Because our study was based on a larger sample and more than one study population, it questions the generality of the findings reported by Calhim *et al.* [11].

In our study populations, extrapair paternity only accounts for about 5% of nestlings [12]–[14], and is low even in experimental situations where females should have the opportunity to copulate with blacker, extrapair males in the vicinity [34]. In general, male pied flycatchers do not guard their mates during the supposed fertile period [35], which is largely the three days preceding the start of egg-laying [36]. During this period they often spend considerable amount of time away from their primary territory to advertise for additional females [12]. The low rate of extrapair paternity can therefore not be explained by a lack of mating opportunities for females. Females prefer blacker males for their parental care [19] and early arriving males for their territories [17]. If females were also to obtain genetic benefits by choosing these males, females paired with inferior males should seek extrapair copulations with these males, and extra-pair paternity should be common. The same argument applies if females seek extrapair copulations only for fertility insurance [20], and black and early-arriving males have more fertile sperm. These hypothetical quality benefits do not seem to apply, since black males are not cuckolded less often than brown males [15], [16], [37]. Hence, we argue that pre-copulatory sexual selection on male sperm quality traits seems unlikely in the pied flycatcher.

The assumption of longer sperm being competitively superior is also not supported by our analysis. Longer sperm did not swim faster, at least not in our standardized test medium, and had a significantly steeper decline in swimming speed within the first 10 min after sampling. A similar result was recently found in the house sparrow *Passer domesticus* [38], and may indicate that longer sperm utilize their energy reserves differently from shorter sperm. Thus, if measured velocity reflects some form of male fertility, longer sperm seem to perform worse than shorter sperm. We will argue that there is little reason to expect a strong positive relationship between sperm competitiveness and sperm length among males in species with low risk of sperm competition, and perhaps not even in those with higher risks either. Typically, between-male variation in sperm size is smaller in species with high risk of sperm competition [7]–[9], which is generally interpreted as an outcome of stabilizing selection imposed by sperm competition. This means that sperm sizes around the population mean should be favored by selection, and there should be little opportunity for directional selection on longer sperm. Similarly, the high between-male variation in sperm length in species with little or no sperm competition implies relaxed selection on the trait. We therefore conclude that there is neither any supportive empirical evidence, nor any consistent theory, to expect pre-copulatory sexual selection on sperm length in passerine birds.
